# Coronary Plaque Vulnerability and Pericoronary Adipose Tissue Inflammation: Emerging Insights from Advanced CT Imaging

**DOI:** 10.3390/medicina62040630

**Published:** 2026-03-26

**Authors:** Botond Barna Mátyás, Imre Benedek, Nóra Rat, Renáta Gerculy, Theodora Benedek

**Affiliations:** 1 Department of Cardiology, “George Emil Palade” University of Medicine, Pharmacy, Science and Technology of Târgu Mureș, 540139 Târgu Mureș, Romania; matyas_botond@yahoo.com (B.B.M.); imre.benedek@umfst.ro (I.B.); gerculy_renata@yahoo.com (R.G.); theodora.benedek@umfst.ro (T.B.); 2 Clinic of Cardiology, Mureș County Emergency Clinical Hospital, 540136 Târgu Mureș, Romania; 3 Center of Advanced Research in Multimodality Cardiac Imaging, CardioMed Medical Center, 540124 Târgu Mures, Romania

**Keywords:** coronary artery disease, cardiac computed tomography, pericoronary adipose tissue, fat attenuation index, CAD-RADS, high-risk plaque features, risk prediction

## Abstract

Cardiovascular emergencies most frequently arise from the sudden destabilization of atherosclerotic plaques. Conventional diagnostic strategies predominantly focus on luminal stenosis, despite the fact that most acute coronary events originate from non-obstructive lesions with high inflammatory activity. Recent advances in cardiac computed tomography (CCT) enable visualization of plaque morphology and surrounding perivascular fat, offering a unique window into coronary inflammation. The fat attenuation index (FAI), derived from pericoronary adipose tissue (PCAT) radiodensity, has emerged as a dynamic imaging biomarker capable of detecting vascular inflammation before clinical events occur. This review summarizes current evidence on the role of PCAT inflammation in plaque vulnerability, its implications for acute cardiovascular presentations, and recent technological innovations—including AI-enhanced analysis and photon-counting CT—that advance risk prediction. Inflammation-based imaging derived from CCT, including PCAT-FAI, has emerged as a promising research tool that may enhance risk stratification in patients presenting with chest pain. These developments signify a shift from purely anatomical assessment toward biological characterization of CAD, potentially transforming prevention and acute care.

## 1. Introduction

Cardiovascular diseases (CVDs) remain the leading global cause of death, accounting for 17.5 million fatalities in 2012, with ischemic heart disease and stroke representing the largest share [1]. Projections indicate that annual CVD-related deaths may rise to 23.6 million by 2030, driven by aging populations and persistent exposure to modifiable risk factors such as hypertension, diabetes, obesity, and smoking [2]. Recent estimates from the 2023 Global Burden of Disease Study again identify ischemic heart disease as a dominant contributor to worldwide mortality, particularly where preventive strategies remain suboptimal [3]. This burden is reflected in Romania, where CVDs were responsible for over half of all deaths in 2022, with ischemic heart disease and stroke greatly exceeding European mortality averages and contributing to the nation’s persistently reduced life expectancy [4].

While advances in cardiac computed tomography (CCT) have strengthened non-invasive evaluation of coronary artery disease (CAD) [5,6,7], reliance on luminal stenosis alone is insufficient, as many acute coronary syndromes (ACS) originate from non-obstructive yet highly inflamed plaques [8,9,10]. Emerging evidence positions vascular inflammation as a central driver of plaque vulnerability, highlighting the need for biologically informed imaging. Radiomic assessment of pericoronary adipose tissue (PCAT), particularly through the fat attenuation index (FAI), offers a sensitive marker of local inflammatory activity that complements anatomical characterization [11,12,13]. Combined with innovations such as CT-derived fractional flow reserve (CT-FFR) and AI-enabled risk platforms, CCT is transitioning from solely an anatomical tool to a modality capable of capturing dynamic aspects of plaque biology, enabling improved risk stratification and personalized prevention strategies [14,15,16,17,18]. Although PCAT-derived biomarkers have demonstrated strong prognostic associations in observational cohorts, their integration into routine clinical pathways remains under investigation.

This article was designed as a narrative review summarizing current evidence on PCAT imaging and its role in coronary inflammation and plaque vulnerability. Relevant literature was identified through searches of major biomedical databases (e.g., PubMed and Scopus), focusing on studies evaluating PCAT attenuation, the FAI, CCT, and imaging biomarkers related to CAD. Priority was given to landmark clinical trials, observational studies, and recent consensus documents to provide an integrated overview of emerging inflammation-based imaging approaches.

## 2. Coronary Plaque Biology and Vulnerability

### 2.1. Plaque Morphology and Composition

**Figure 1:**
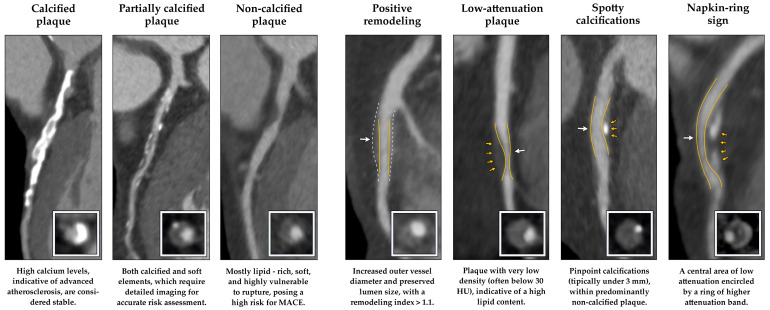
Representative CCT images illustrating plaque composition—CP, PCP or mixed plaques, and NCP—and HRP characteristics associated with adverse cardiac outcomes. Yellow lines outline vessel contours; arrows indicate high-risk features and remodeling; dashed lines show the reference contour.

Coronary atherosclerosis encompasses a spectrum of plaque phenotypes, each with distinct structural and clinical implications. Plaques are broadly categorized according to the degree of calcification into calcified plaques (CP), partially calcified plaques (PCP), and non-calcified plaques (NCP), as shown in Figure 1. CPs are typically associated with more advanced but mechanically stable disease, reflecting a chronic reparative process that tends to limit the risk of rupture [19,20]. PCPs, by contrast, contain both calcific and lipid-rich components and often represent a transitional form of atherosclerosis whose complexity may require advanced imaging for accurate characterization [19,21].

NCPs are of particular clinical concern. These lesions are enriched in lipid material and inflammatory cells and commonly exhibit features of thin-cap fibroatheroma (TCFA), defined by a substantial necrotic core and a fragile fibrous cap. Such plaques are predisposed to fissuring or rupture even in the absence of significant luminal narrowing [22,23,24,25]. Numerous invasive and non-invasive imaging studies have shown that NCPs—not obstruction severity—account for a large share of myocardial infarctions [24,26]. Furthermore, these high-risk phenotypes frequently occur in younger individuals and in patients with zero coronary artery calcium (CAC) scores, underscoring the limitations of CAC as a standalone indicator of risk [27,28,29].

Although CAC remains an established marker of cumulative plaque burden and long-term cardiovascular risk [9,30,31], its absence does not preclude the presence of vulnerable NCPs. This limitation has become increasingly evident in symptomatic patients with CAC = 0, in whom biologically active plaques may still be present [12,28,32]. Modern data also highlight that statin therapy—while stabilizing plaques overall—can increase coronary calcification, complicating interpretation of CAC trajectories [33,34,35,36]. These nuances illustrate why understanding plaque composition, not only burden, is essential for evaluating short-term risk.

### 2.2. High-Risk Plaque Imaging Markers

Beyond morphologic classification, CCT enables identification of a series of imaging signatures that reflect underlying plaque biology and instability. These so-called high-risk plaque (HRP) markers include positive remodeling (PR), low-attenuation plaque (LAP), spotty calcifications (SC), and the napkin-ring sign (NRS), as shown in Figure 1. PR—defined as a remodeling index > 1.1—suggests compensatory vessel expansion in response to inflammation. LAP, characterized by attenuation < 30 HU, corresponds to lipid-laden necrotic cores, whereas SC represents small punctate calcifications (<3 mm) scattered within the plaque. The NRS, a circumferential high-attenuation rim surrounding a low-attenuation core, is particularly specific for TCFA and has consistently been associated with impending plaque rupture [37,38,39].

**Figure 2:**
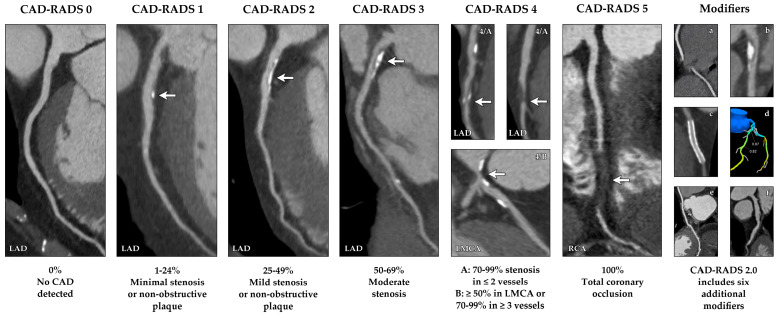
CAD-RADS 2.0 categories and modifiers. CCT examples demonstrating varying degrees of coronary artery stenosis based on the CAD-RADS classification. White arrows indicate the location and severity of coronary stenosis. Modifiers include: (**a**) non-diagnostic/limited evaluability; (**b**) HRP; (**c**) presence of stents; (**d**) ischemia assessment via FFR-CT; (**e**) bypass grafts; (**f**) exceptions such as LAD ectasia.

These HRP markers have strong prognostic value. Their presence predicts major adverse cardiovascular events (MACE) independently of stenosis severity [37,40,41]. Consequently, HRP detection has become a crucial component of modern CCT interpretation, prompting the development of structured frameworks such as CAD-RADS 2.0 (as shown in Figure 2 and Table 1), which integrates HRP characteristics and plaque burden alongside stenosis grading [42,43,44]. This multiparametric approach allows for a more biologically informed risk profile, facilitating earlier intervention in patients harboring vulnerable plaque features even when luminal obstruction is modest.

### 2.3. Non-Obstructive CAD as a Cause of Acute Events

While obstructive CAD remains an established predictor of adverse outcomes, accumulating evidence demonstrates that non-obstructive lesions with HRP features represent a major source of acute coronary events. CCT provides excellent negative predictive value for ruling out significant stenosis [45,46], and patients with entirely normal CCT findings typically exhibit excellent long-term prognosis [47,48]. However, those with non-obstructive plaque—defined as <50% luminal narrowing—experience significantly higher rates of MACE compared with individuals without any detectable atherosclerosis [49,50]. Importantly, non-obstructive plaques displaying LAP, PR, or NRS remain vulnerable to rupture despite their limited impact on luminal caliber [51,52].

Large observational registries reinforce this paradigm shift. The CONFIRM registry demonstrated a graded increase in mortality with increasing stenosis severity [53], yet studies such as ICONIC revealed that non-obstructive plaques with HRP carry a risk of future events comparable to that of obstructive plaques without vulnerability markers [51,54]. Findings from the PROMISE trial further highlight that HRP features confer independent prognostic significance, increasing event rates up to 2.7-fold in patients with <50% stenosis [51,52,55].

The PARADIGM registry advanced this concept by integrating plaque volume, composition, and progression into a longitudinal risk model, underscoring the need to identify biologically active plaques before they evolve into clinically manifest events [49,56]. Early recognition of these high-risk non-obstructive lesions enables timely preventive strategies, including intensive lipid-lowering therapy and lifestyle modification, ultimately aiming to avert ACS before mechanical obstruction occurs. Key clinical studies supporting these findings are summarized in Table 2.

**Table 2:** Major clinical studies evaluating coronary plaque vulnerability using CCT.

Study	Population	Plaque Features	Main Findings	Clinical Implication
Motoyama et al., 2009 [40]	1059 patients with suspected CAD undergoing CCT	PR, LAP	Combination of PR and LAP strongly predicted future ACS events	First prospective evidence linking CCT plaque morphology to ACS risk
Motoyama et al., 2015 [57]	895 patients with CCT and follow-up	PR, LAP, SC, NRS	High-risk plaque features were associated with significantly higher incidence of ACS during follow-up	Established prognostic importance of HRP features
Maurovich-Horvat et al., 2012 [51]	Patients undergoing CCT for suspected CAD	NRS	NRS strongly associated with advanced and vulnerable plaques	Provided imaging signature of TCFA
Otsuka et al., 2013 [38]	Patients evaluated by CCT with clinical follow-up	NRS	Presence of NRS significantly predicted future acute coronary syndrome	Reinforced NRS as a marker of plaque vulnerability
Williams et al., SCOT-HEART trial 2020 [58]	Multicenter randomized trial cohort	NCP and LAP	Non-calcified plaque burden predicted MI independent of stenosis severity	Shifted focus from stenosis to plaque biology
Lu et al., 2021 [39]	Patients undergoing CCT for CAD evaluation	PR, LAP, SC, NRS	HRP features significantly improved prediction of ACS events	Supports multiparametric plaque assessment
Bauer et al., 2009 [52]	Patients undergoing CCT and myocardial perfusion imaging	NCP burden	Non-calcified plaque burden correlated with ischemia	Early demonstration of prognostic plaque features
Virmani et al., 2006 [23]	Pathology-based plaque analysis	TCFA	TCFA identified as the primary substrate of plaque rupture	Histopathologic foundation for imaging markers
Salem et al., 2023 [37]	Patients undergoing CCT with plaque characterization	HRP features and TCFA	CCT features correlate with invasive imaging markers of vulnerability	Validates CT-based plaque characterization

## 3. Imaging Inflammation: PCAT, EAT, and the Fat Attenuation Index

### 3.1. Biological Rationale

**Figure 3:**
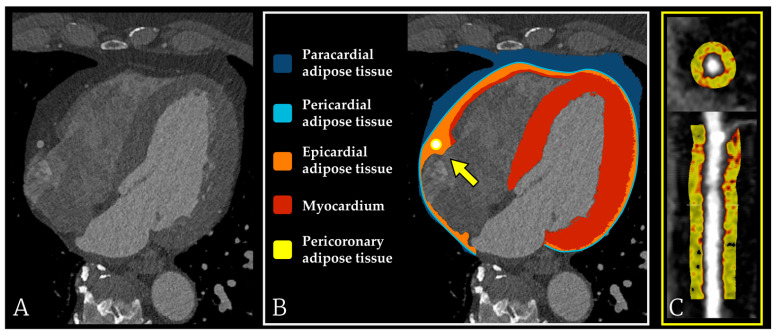
Cardiac adipose tissue compartments on CCT. (**A**) CCT cross-section without adipose tissue delineation. (**B**) Schematic depiction of paracardial, pericardial, epicardial, myocardial, and pericoronary adipose tissue (PCAT), with the coronary artery surrounded by PCAT; the arrow indicates the PCAT region analyzed. (**C**) Axial and longitudinal coronary reconstructions highlighting PCAT (yellow) used for radiomic analysis and FAI quantification.

Over the past decade, a growing body of evidence has repositioned epicardial and pericoronary adipose tissue from passive fat depots to metabolically active organs with direct influence on coronary biology. Epicardial adipose tissue (EAT) can be evaluated using several imaging modalities, including echocardiography, CCT, and cardiac magnetic resonance imaging, each providing complementary information on its anatomical distribution and biological role. Early echocardiographic studies established EAT thickness as a non-invasive marker of cardiometabolic risk, while modern CCT techniques allow detailed volumetric and compositional characterization of EAT and PCAT [59]. EAT, situated beneath the visceral pericardium and in direct contact with the myocardium, is uniquely vascularized by branches of the coronary arteries, allowing an uninterrupted exchange of bioactive molecules between adipocytes and cardiac structures. In contrast, PCAT surrounds the coronary vessels themselves, forming what has been termed the “fourth layer” of the arterial wall (Figure 3). Its strategic location permits intense paracrine and vasocrine communication with the vascular endothelium, smooth muscle cells, and infiltrating immune cells [12,60].

Physiologically, these adipose layers secrete a balanced profile of adipokines and cytokines—ranging from adiponectin and other protective mediators to factors promoting vasodilation and antioxidative homeostasis. However, metabolic dysfunction shifts this equilibrium toward a pro-inflammatory, pro-atherogenic state. Obesity, diabetes, and metabolic syndrome promote adipocyte hypertrophy, oxidative stress, and macrophage infiltration, transforming both EAT and PCAT into potent sources of inflammatory mediators such as IL-6, TNF-α, MCP-1, and profibrotic signaling molecules [60,61,62]. Because PCAT lies directly adjacent to the coronary adventitia, inflammatory activation within this tissue mirrors—and amplifies—pathological processes occurring in the coronary artery wall, contributing to endothelial dysfunction and plaque fragility. An overview of key clinical studies is provided in Table 3.

**Table 3:** Major studies evaluating PCAT attenuation and the FAI using CCT.

Study	Population	PCAT/FAI Parameters	Main Findings	Clinical Implication
Antonopoulos et al., 2017 [12]	Experimental and translational analysis of coronary inflammation	PCAT attenuation	Demonstrated that coronary inflammation modifies PCAT attenuation detectable on CCT	Established the biological basis for FAI as a non-invasive marker of coronary inflammation
Oikonomou et al., CRISP-CT, 2018 [18]	Large prospective cohort undergoing CCT	PCAT attenuation	Increased FAI independently predicted cardiac mortality and major cardiovascular events	Landmark validation of FAI as a prognostic biomarker
Goeller et al., 2019 [33]	Patients undergoing serial CCT imaging	Longitudinal changes in PCAT attenuation	Changes in PCAT attenuation correlated with coronary plaque burden progression	Demonstrated the potential of FAI-related metrics for disease monitoring
Lin et al., 2021 [34]	Cross-sectional cohort of patients with varying CAD severity	PCAT attenuation	PCAT attenuation differed significantly across stages of CAD	Supported diagnostic value of PCAT-derived inflammation markers
Van Rosendael et al., 2024 [35]	Patients undergoing CCT with long-term follow-up	PCAT attenuation	Elevated PCAT attenuation predicted long-term cardiovascular outcomes	Confirmed the prognostic relevance of coronary inflammation imaging
Goeller et al., 2018 [63]	Patients with acute coronary syndrome and stable CAD	PCAT attenuation surrounding culprit lesions	Higher PCAT attenuation associated with high-risk plaques and ACS	Demonstrated the link between local vascular inflammation and plaque vulnerability
Dai et al., 2020 [64]	Patients undergoing serial CCT after statin therapy	Serial changes in FAI	FAI decreased following statin therapy, suggesting reduced coronary inflammation	Supports the use of FAI for monitoring therapeutic response
Bao et al., 2022 [65]	Patients with psoriasis and matched controls	FAI-derived coronary inflammation	Elevated FAI reflected increased coronary inflammation in systemic inflammatory disease	Expanded clinical relevance of PCAT-derived markers
Oikonomou et al., 2021 [66]	Translational study of coronary inflammation quantification	PCAT measurement	Proposed standardized methods for coronary inflammation imaging	Important step toward clinical implementation of FAI
Chan et al., ORFAN cohort, 2024 [67]	Multicenter longitudinal cohort without obstructive CAD	PCAT-derived FAI	Coronary inflammation predicted cardiovascular events even without obstructive disease	Highlighted the role of PCAT imaging in early risk stratification
Mátyás et al., 2023 [68]	Patients following COVID-19 infection	PCAT-derived FAI	Elevated FAI indicated persistent coronary inflammation and increased plaque vulnerability risk	Demonstrated applicability of FAI in post-viral inflammatory states
Gerculy et al., 2025 [69]	Patients with atrial fibrillation compared with controls	PCAT-derived FAI	Increased coronary inflammation associated with atrial fibrillation development	Suggests interaction between coronary inflammation and atrial arrhythmogenesis

### 3.2. Technical Basis of PCAT Measurement

Advances in cardiac CT have enabled precise quantification of both EAT and PCAT, but PCAT has gained particular prominence due to its close relationship with coronary inflammation. PCAT attenuation is measured within a radiodensity range of −190 to −30 Hounsfield units (HU), allowing radiologists to assess tissue composition and inflammatory state with high reproducibility [12]. The FAI refines this assessment by capturing subtle, spatial changes in PCAT radiodensity related to shifts in adipocyte size, intracellular lipid/water content, and microvascular remodeling.

**Figure 4:**
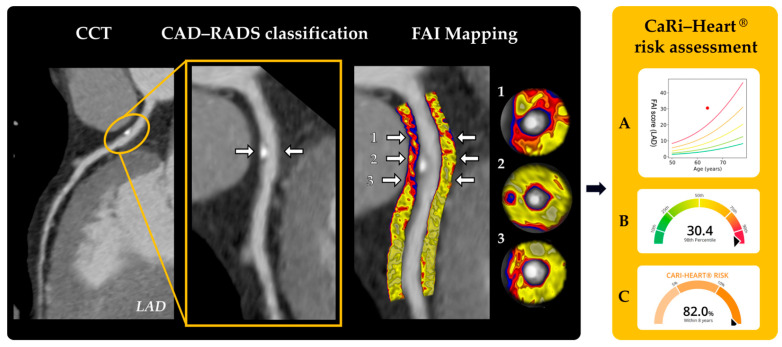
Workflow for PCAT analysis and FAI quantification from CCT. In the black panel, CCT demonstrates lesion localization, with arrows indicating the site of coronary plaque. CAD-RADS-based plaque classification is followed by circumferential PCAT segmentation (−190 to −30 HU); numbered arrows (1–3) indicate segment-specific perivascular regions where FAI is sampled along the vessel. Color-coded FAI mapping illustrates the spatial heterogeneity of pericoronary inflammation, with corresponding cross-sectional views (1–3) showing local FAI distribution used for quantitative analysis. In the yellow CaRi-Heart® panel, derived FAI metrics are translated into clinical risk assessment: (**A**) the red dot represents the patient-specific FAI value relative to age-adjusted risk curves; (**B**) percentile-based inflammation score; (**C**) individualized cardiac risk estimate.

An important conceptual distinction has emerged between peri-arterial and peri-plaque PCAT. While early studies focused on PCAT surrounding proximal vessel segments—most often the RCA—newer radiomic approaches now analyze PCAT adjacent to specific plaques, where local inflammatory activity may be more pronounced [70,71]. This lesion-specific evaluation increases diagnostic sensitivity, particularly in patients with focal non-calcified or partially calcified plaques that carry high rupture risk (Figure 4). The recognition of PCAT as an imaging biosensor of coronary inflammation has been endorsed by recent expert consensus statements, though standardized protocols for routine clinical use are still under development [72].

### 3.3. Validation Studies and Clinical Translation

The clinical relevance of PCAT-derived inflammation metrics was firmly established by the landmark CRISP-CT study, which demonstrated that elevated FAI around the proximal RCA strongly predicts cardiac mortality [18]. Individuals with FAI values above −70.1 HU exhibited a nearly sevenfold increased risk of fatal cardiac events, independent of plaque burden, CAC scores, and traditional risk factors [73]. These findings confirmed that FAI captures a distinct and clinically meaningful dimension of cardiovascular risk: vascular inflammation. However, it should be noted that current evidence supporting FAI is primarily derived from observational cohorts, and randomized trials evaluating FAI-guided management strategies are not yet available.

The role of PCAT-derived biomarkers should be interpreted in the context of other inflammatory and imaging markers. Systemic biomarkers such as high-sensitivity C-reactive protein (hsCRP) reflect global inflammation and have demonstrated prognostic value but lack spatial specificity for coronary disease [74]. Molecular imaging with PET and ^18F-sodium fluoride (NaF) PET can identify metabolically active or microcalcified plaques associated with vulnerability [75], while invasive techniques such as intravascular ultrasound (IVUS) and optical coherence tomography (OCT) provide high-resolution plaque characterization and have been linked to future coronary events in studies such as PROSPECT [76]. In contrast, the FAI derived from CCT enables non-invasive assessment of local pericoronary inflammation and complements anatomical plaque evaluation [18,66,72].

**Figure 5:**
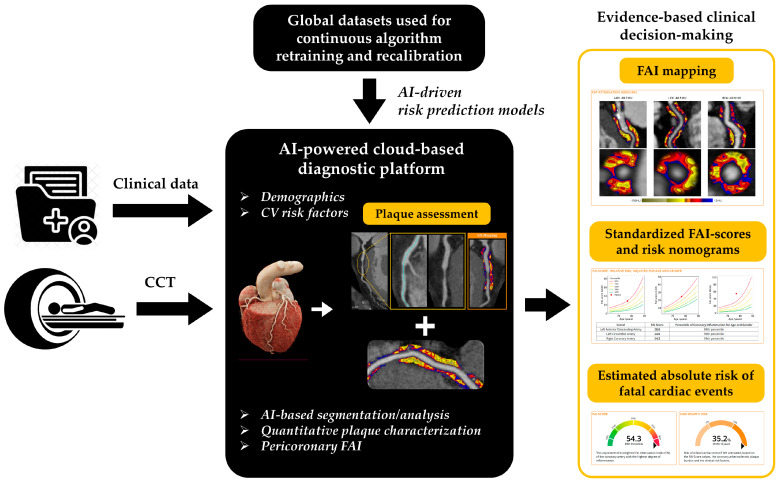
Integrated workflow of an AI-powered cloud-based platform for cardiovascular risk prediction. Clinical data and CCT images are processed using automated segmentation and analysis tools to extract key biomarkers, including plaque characteristics and the pericoronary FAI. These parameters are combined with international datasets to recalibrate risk prediction algorithms. The system generates harmonized FAI maps and individualized risk scores, allowing estimation of absolute risk for fatal cardiac events and supporting informed clinical decision-making [11,12,73].

Subsequent research has validated FAI in a wide range of clinical settings (Figure 5). Elevated FAI correlates with CAD severity, the presence of high-risk plaque morphology, and future ACS occurrence—even in patients with non-obstructive disease or low CAC scores [34,35,63]. Unlike calcification, which reflects cumulative disease burden, or HRP markers, which indicate structural vulnerability, FAI quantifies current, modifiable inflammatory activity. Its dynamic nature is particularly relevant for therapy monitoring. Studies show that statins and biologic anti-inflammatory treatments significantly reduce FAI values, consistent with decreases in systemic and vascular inflammation [64,65]. This responsiveness underscores the potential use of FAI in guiding treatment intensity and tracking therapeutic success.

Beyond atherosclerosis, PCAT attenuation has proven valuable in characterizing inflammation in systemic inflammatory conditions. Persistent elevation of FAI has been observed in individuals recovering from SARS-CoV-2 infection, suggesting ongoing coronary inflammation despite minimal or absent anatomical stenosis [68,77,78]. Evidence from clinical and imaging studies indicates that elevated FAI can identify inflamed, rupture-prone plaques even in young adults with post-COVID presentations, highlighting the potential role of coronary inflammation in post-viral cardiovascular risk [79]. Reductions in FAI after biologic therapy have also been demonstrated in psoriasis, further supporting its use as a real-time vascular inflammation biomarker [80,81].

### 3.4. Radiomics and Artificial Intelligence (CaRi-Heart^®^)

The clinical adoption of PCAT-based metrics has accelerated with the emergence of AI-powered radiomics platforms. Tools such as CaRi-Heart^®^ automate segmentation of PCAT, extract textural and functional radiomic variables, and integrate them with plaque characteristics and clinical data to generate individualized inflammation and risk scores. These systems harmonize CCT images, apply validated algorithms calibrated on international datasets, and produce color-coded FAI maps and absolute risk estimates for adverse cardiovascular events.

By combining anatomical, biological, and computational assessments, radiomics platforms bridge the gap between plaque morphology and its inflammatory microenvironment. This capability enhances risk prediction beyond conventional CCT markers and supports personalized preventive strategies. As regulatory pathways advance and standardization improves, automated PCAT analysis is expected to become a routine component of CCT interpretation, reshaping clinical pathways for assessing coronary inflammation and plaque vulnerability.

## 4. Coronary Inflammation in Cardiovascular Emergencies

### 4.1. Acute Coronary Syndromes (Plaque Rupture/Erosion)

ACS arise predominantly from the sudden destabilization of atherosclerotic plaques rather than progressive luminal obstruction. Over the past decade, imaging studies have shown that perivascular inflammation—captured through the FAI—is closely linked to the biological processes that render plaques susceptible to rupture or erosion. Elevated FAI reflects inflammatory remodeling of pericoronary adipose tissue, which often surrounds plaques with a large necrotic core, thin fibrous cap, or positive remodeling—features typical of vulnerable lesions implicated in ACS [52,67]. This relationship has been validated across diverse populations, demonstrating that increased PCAT attenuation serves as an early warning signal of plaque instability and strongly predicts coronary events independent of stenosis severity or CAC burden [18,64,70].

Clinical imaging evidence further supports the central role of inflammation-driven plaque destabilization in ACS. In patients presenting with acute myocardial infarction—including younger individuals and those with recent systemic inflammatory conditions—CCT angiography has identified non-calcified or mixed plaques in the proximal coronary segments that exhibit high-risk plaque features despite only moderate or sub-obstructive stenosis [82,83]. In these cases, lesion-specific PCAT analysis revealed markedly elevated FAI values, indicating intense localized vascular inflammation. Subsequent ICA confirmed the culprit lesions, while intravascular imaging with optical coherence tomography frequently demonstrated plaque erosion or rupture with superimposed thrombus [79]. Across multiple cases, concordance between vulnerable plaque morphology, high perivascular inflammatory burden, and acute clinical presentation underscores the mechanistic link between inflammation and plaque failure.

Taken together, these observations reinforce that ACS frequently originates from lesions whose danger lies not in anatomical narrowing but in inflammatory activation. FAI may complement traditional plaque characterization by providing information on pericoronary inflammation associated with plaque vulnerability.

### 4.2. Atrial Fibrillation as an Inflammatory Emergency

Atrial fibrillation (AF) is increasingly recognized as a disorder in which inflammation contributes to both arrhythmia initiation and maintenance. EAT—a metabolically active visceral fat depot closely adherent to the myocardium—plays a central role in this process through its secretion of pro-inflammatory and profibrotic mediators such as IL-6, TNF-α, and MCP-1 [84]. These adipocytokines diffuse directly into the atrial myocardium, promoting structural remodeling, fibrosis, and electrical instability.

Advances in CCT now allow clinicians to assess not only the volume of EAT but also its inflammatory state using radiomic biomarkers such as FAI. In our work, AF patients displayed significantly higher EAT volumes compared with non-AF controls, supporting the link between epicardial fat burden and atrial susceptibility to arrhythmogenic remodeling [85]. When coronary inflammation was quantified using perivascular FAI, AF patients exhibited elevated inflammatory signals, particularly around the left coronary arteries—an observation that suggests a convergence between coronary and atrial inflammatory pathways [69].

These findings have important clinical implications. Since AF heightens the risk of stroke, heart failure, and hemodynamic compromise, early recognition of inflammation-driven arrhythmogenic substrates may refine patient selection for rhythm-control interventions and guide preventive strategies. Moreover, assessing both EAT volume and perivascular inflammation may help identify individuals at higher risk for AF recurrence after cardioversion or ablation, enabling a more personalized management approach.

### 4.3. Post-COVID Patients Presenting with Chest Pain or ACS-like Symptoms

COVID-19 has introduced new challenges in evaluating patients presenting with chest pain or possible ACS. Even months after infection, many individuals exhibit persistent vascular inflammation despite minimal or absent coronary stenosis. Our post-SARS-CoV-2 cohort demonstrated significantly elevated PCAT-FAI values in recovered patients compared with uninfected controls, consistent with prolonged coronary immune activation [68,77]. Similar findings have been reported in independent CT studies, including non-contrast scans showing increased peri-coronary fat density, particularly around the RCA [78,86].

This persistent inflammatory phenotype complicates emergency evaluation. COVID-related myocardial symptoms—such as chest pain, dyspnea, or biomarker elevation—may mimic ACS, yet the underlying mechanism may involve microvascular dysfunction, endothelial injury, or inflammation rather than plaque rupture. In such contexts, traditional tools like ECG, troponin, or even angiography may fail to distinguish true ACS from myocarditis-like or endothelial inflammatory presentations. FAI thus offers a unique advantage: it identifies localized vascular inflammation that may indicate heightened risk even when anatomical imaging appears unremarkable.

Case reports, including the STEMI example described earlier, highlight how COVID-related inflammation may accelerate plaque destabilization, particularly in young or low-risk individuals [79]. Beyond COVID-19, similar FAI elevations have been noted in systemic inflammatory diseases such as psoriasis, where biologic therapy resulted in significant reductions in perivascular inflammation [80,81,87]. These findings suggest that PCAT-based inflammation imaging may provide valuable insight into the cardiovascular consequences of systemic immune activation.

## 5. CT-Based Preventive and Emergency Risk Stratification

### 5.1. Role of CAC in Emergency Pathways

**Figure 6:**
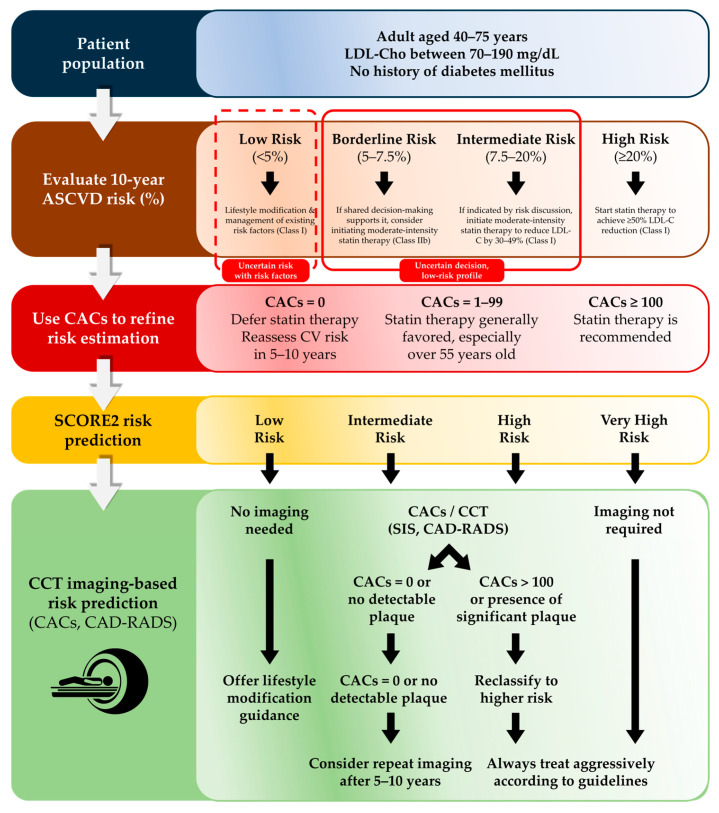
Contemporary use of CAC in primary prevention and its role in stepped risk assessment. CAC is useful in intermediate-risk and selected borderline-risk adults when risk decisions are unclear and no major risk enhancers are present. It may also be considered in low-risk individuals with significant risk factors. Statin therapy can be deferred if CAC = 0, favored if CAC = 1–99, and recommended if CAC ≥ 100. Adapted from AHA/ACC guidelines [88]. In low-risk groups, CAC is not routinely recommended, though ~4% may still have significant CAC [89].

CAC remains one of the most widely used tools for initial cardiovascular risk assessment and is particularly valuable in emergency pathways where rapid stratification is essential. Derived from non-contrast CT, CAC quantifies total calcified plaque burden and provides a straightforward, reproducible metric that enhances traditional clinical risk estimators such as SCORE-2 and ASCVD models [88,89,90,91,92]. In intermediate-risk individuals or those with borderline indications for statin therapy, CAC helps refine preventive decisions: a score of zero may support deferral of pharmacologic therapy, while values ≥ 1, and especially ≥100, significantly favor treatment initiation (Figure 6).

Despite its widespread use, CAC is fundamentally a static marker reflecting cumulative plaque burden rather than current disease activity. It does not capture early non-calcified lesions, inflammatory remodeling, or dynamically evolving plaque vulnerability—particularly relevant in emergency settings where ACS often originates in plaques that contain little or no calcium. Younger patients, women, and individuals from certain ethnic backgrounds may experience significant atherosclerotic risk despite a CAC of zero, highlighting an important limitation in relying exclusively on calcification for risk exclusion [89,90,91,93,94,95].

For these reasons, CAC serves as a strong long-term prognostic indicator but must be interpreted cautiously when evaluating acute symptoms or short-term risk. Integration with anatomical CCT, plaque characterization, and inflammation imaging can therefore provide a more comprehensive emergency assessment.

### 5.2. CCT in Asymptomatic and Symptomatic Risk Triage

**Figure 7:**
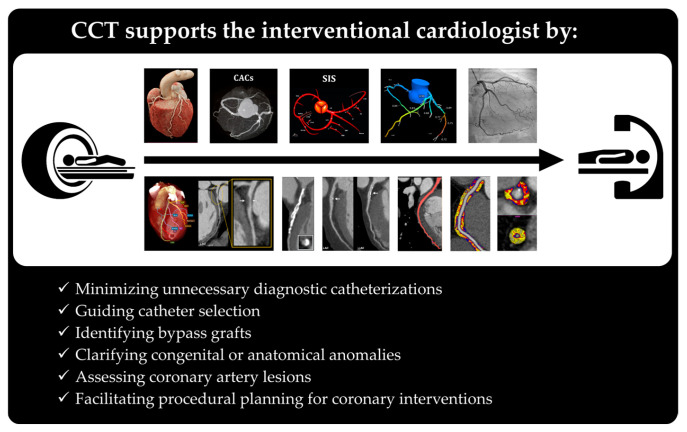
Role of CCT in supporting interventional cardiology. CCT aids interventional cardiologists by reducing unnecessary diagnostic catheterizations, guiding catheter selection, identifying grafts, clarifying anomalies, assessing coronary lesions, and supporting procedural planning.

CCT offers a more detailed visualization of coronary anatomy than CAC alone, enabling identification of both obstructive and non-obstructive plaques, quantification of total plaque burden, and detection of high-risk plaque features. Its diagnostic strength was demonstrated in major trials: SCOT-HEART showed that CCT-guided evaluation improved diagnostic precision and significantly reduced subsequent myocardial infarction, while PROMISE confirmed that anatomical CT assessment outperformed conventional functional testing for predicting MACE [20,96]. Cost-effectiveness analyses further indicate that CCT reduces unnecessary invasive coronary angiography (ICA) by up to 77% (Figure 7), limiting patient exposure to procedural risks and decreasing healthcare utilization [97,98].

However, the role of CCT differs substantially between symptomatic and asymptomatic populations. While symptomatic patients clearly benefit from detailed anatomical assessment, asymptomatic individuals show more heterogeneous results. The CONFIRM registry found that adding CCT to traditional risk models did not significantly enhance prognostication in asymptomatic cohorts, reinforcing CAC as the preferred first-line imaging modality in population screening scenarios [53,99]. Ongoing investigations, including SCOT-HEART 2, aim to clarify whether CCT may still serve a role in selected high-risk individuals without symptoms [8,58,100].

Importantly, a CAC score of zero does not guarantee absence of clinically relevant disease. Up to 46% of asymptomatic adults in large cohort studies exhibited non-calcified or mixed plaques on CCT, including lesions with high-risk features [101]. This underscores the biological distinction between calcification and inflammation: vulnerable plaques often remain entirely non-calcified, and inflammatory activity—as captured by PCAT-FAI—may be elevated even when CAC is zero [57,102]. Thus, in both preventive and emergency settings, CCT combined with inflammation imaging offers a more nuanced assessment of near-term risk.

Technological progress continues to improve the utility and safety of CCT. Low-dose acquisition techniques, iterative reconstruction algorithms, and emerging photon-counting CT (PCCT) systems significantly reduce radiation exposure while enhancing image quality, expanding the feasibility of CCT in triage pathways [103,104,105].

### 5.3. Dynamic Monitoring of Treatment Response

The ability to monitor disease progression and therapeutic response is a major advantage of CT-based imaging beyond conventional risk estimation. While systemic inflammatory markers such as CRP provide global estimates of risk, they lack vessel-level specificity. In contrast, CCT-derived metrics—including PCAT-FAI, HRP features, and volumetric plaque quantification—offer a localized and dynamic view of atherosclerotic biology [18,106].

Lipid-lowering therapy illustrates the value of this approach. Longitudinal imaging studies, including our own work, have demonstrated that patients treated with high-intensity statins show progressive transformation of plaque composition: increases in calcified components, reductions in non-calcified plaque volume, and sustained decreases in PCAT attenuation—a pattern consistent with reduced inflammatory activity and plaque stabilization [16,107]. These imaging changes parallel improved clinical outcomes and may help identify individuals who remain at residual inflammatory risk despite adequate lipid control.

Anti-inflammatory therapies provide another avenue where dynamic imaging is particularly informative. The CANTOS trial established that targeting IL-1β pathways reduces recurrent cardiovascular events independently of LDL levels, supporting inflammation as a therapeutic target [108]. Emerging biologics—including rilonacept and ziltivekimab—are now being evaluated for similar effects, and radiomic biomarkers such as PCAT-FAI may serve as sensitive tools to track their impact on vascular inflammation [109].

In the emergency context, the ability to monitor plaque stability over time may help identify patients at imminent risk of ACS recurrence and guide the intensity of preventive therapy. As CCT technologies and AI-enabled analytics continue to advance, routine dynamic monitoring of atherosclerosis may become an integral component of personalized cardiovascular care.

## 6. Emerging Technologies Transforming Emergency Cardiology

### 6.1. CT-FFR for Functional Assessment of Intermediate Lesions

Rapid advances in cardiovascular imaging are reshaping how clinicians evaluate patients at risk of acute coronary events. Traditional diagnostic pathways relied heavily on anatomical stenosis assessment, yet a growing body of evidence demonstrates that functional ischemia, plaque inflammation, and subtle morphologic vulnerability all contribute to the development of ACS. New imaging technologies—including non-invasive CT-FFR, PCCT, and AI-driven risk prediction tools—now offer a more comprehensive assessment of coronary biology and physiology, ultimately improving triage decisions in emergency cardiology.

CT-FFR has emerged as an important complement to CCT, providing a non-invasive physiological assessment of coronary lesions. By applying computational fluid dynamics or machine-learning-based algorithms to standard CCT datasets, CT-FFR estimates the pressure gradient across a stenosis without the need for vasodilator stress or invasive catheterization [110]. Large multicenter trials—including NXT, PLATFORM, and ADVANCE—have consistently shown that CT-FFR significantly improves diagnostic accuracy for identifying hemodynamically significant stenoses, outperforming anatomical CCT alone [111,112,113].

The integration of CT-FFR into emergency diagnostic pathways has practical implications: it markedly reduces unnecessary ICA. In the PLATFORM study, CT-FFR-guided strategies resulted in a 61% reduction in invasive procedures that turned out to have no obstructive disease, while maintaining excellent safety and clinical outcomes [112]. Furthermore, meta-analyses confirm high per-vessel sensitivity (80–90%) and specificity (75–85%), solidifying CT-FFR as a robust gatekeeper to ICA in patients presenting with intermediate stenoses or equivocal symptoms [114].

As computational algorithms become faster and more automated, CT-FFR can be generated within minutes, making it increasingly suitable for real-time decision support in emergency chest pain units. Its ability to identify flow-limiting lesions early may prevent unnecessary hospital admissions and streamline care for patients at imminent risk of plaque rupture.

### 6.2. Photon-Counting CT

PCCT represents a major technological leap in cardiovascular imaging. Unlike conventional energy-integrating detectors, PCCT directly counts individual photons and measures their energy levels, enabling superior spatial resolution, reduced electronic noise, and enhanced tissue differentiation [115]. In coronary imaging, these improvements translate to clearer visualization of stented segments, reduced blooming artifacts from calcification, and improved detection of HRP features such as low-attenuation cores and microcalcifications [116].

PCCT also offers substantial radiation dose reductions—often exceeding 40%—without compromising diagnostic quality [117]. This is particularly valuable in emergency settings, where high throughput and repeated imaging are common. Early clinical studies demonstrate that PCCT identifies HRP characteristics with greater precision than standard CT systems, suggesting a potential role in detecting vulnerable plaques before they precipitate ACS [118].

Moreover, spectral imaging capabilities allow material-specific reconstructions, improving contrast-to-noise ratios and facilitating differentiation between iodine, calcium, and lipid components within plaques. As PCCT becomes more widely available, its enhanced spatial and spectral abilities are expected to advance emergency risk stratification by more accurately characterizing plaque biology in symptomatic patients.

### 6.3. AI-Enhanced Risk Prediction

**Figure 8:**
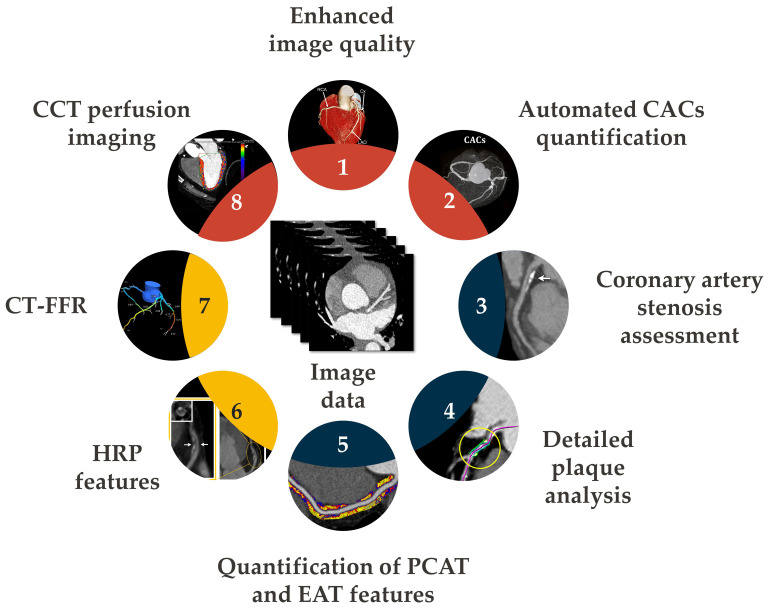
AI is increasingly integrated into CCT workflows, enhancing the diagnostic process for CVDs. AI-based applications support tasks such as automated enhancement of image quality, coronary calcium quantification, stenosis grading, plaque analysis, and measurement of PCAT and EAT. They also facilitate non-invasive functional assessments, including CT-derived FFR and myocardial perfusion evaluation.

Artificial intelligence (AI) is transforming cardiovascular imaging by enabling rapid, fully automated extraction of anatomical, functional, and radiomic biomarkers (Figure 8). Machine-learning algorithms now support plaque segmentation, quantification of total plaque burden, identification of HRP features, measurement of pericoronary fat attenuation, and generation of CT-FFR values—all of which contribute essential information for emergency assessment [119,120].

A landmark advancement in this field is the ORFAN dataset, encompassing over 250,000 individuals with long-term clinical follow-up [67]. This unprecedented resource has enabled the development of AI-driven risk models that integrate plaque characteristics, PCAT radiomics, and clinical variables to predict cardiac mortality with high precision [73,106]. Emerging radiomic signatures derived from perivascular fat have demonstrated the ability to detect subclinical inflammation, differentiate plaque phenotypes, and identify patients with imaging features associated with increased risk of future ACS on conventional imaging [121].

AI-based predictive algorithms are now being tested for real-time decision support in emergency departments. These tools can rapidly flag high-risk imaging patterns, triage patients with chest pain more effectively, and potentially forecast short-term events such as plaque rupture or arrhythmic instability. Early evidence suggests that combining radiomics with deep learning models may outperform traditional risk scores and even expert interpretation in predicting acute cardiovascular events [122,123].

## 7. Clinical Implications and Proposed Diagnostic Algorithm

The growing recognition that acute coronary events frequently arise from biologically active, non-obstructive plaques necessitates a reassessment of current diagnostic strategies for patients presenting with chest pain. Conventional emergency pathways are primarily designed to detect flow-limiting stenoses or ongoing myocardial injury, yet these approaches often fail to identify inflammatory plaque phenotypes that predispose to sudden destabilization. Consequently, incorporating imaging biomarkers that capture both structural and biological aspects of coronary disease is essential for improving early risk stratification.

An integrated CT-based diagnostic framework combining CAC, CCT, and PCAT-FAI has been proposed as a potential strategy to enhance biological risk stratification. CAC remains a valuable first-line tool due to its rapid acquisition, reproducibility, and robust prognostic value for long-term risk assessment. However, its inability to detect non-calcified plaques or active inflammatory processes limits its utility in acute clinical settings. CCT extends risk evaluation by enabling detailed assessment of plaque burden, morphology, and high-risk features, while PCAT-FAI introduces a biological dimension by directly reflecting local vascular inflammation surrounding the coronary arteries.

In future clinical workflows, this multimodal approach may potentially be implemented in a stepwise manner. In patients with low to intermediate clinical risk and non-diagnostic initial testing, CAC can serve as an initial screening tool. If CAC is positive or clinical suspicion persists despite a score of zero, CCT should be performed to assess coronary anatomy and plaque characteristics. The addition of PCAT-FAI allows clinicians to distinguish between ischemic presentations driven by hemodynamically significant lesions and inflammatory presentations associated with vulnerable, rupture-prone plaques. This distinction is particularly relevant in populations where traditional markers underestimate risk, including younger patients, women, and individuals recovering from systemic inflammatory conditions such as COVID-19.

From a research perspective, the potential integration of PCAT-FAI into emergency diagnostic pathways has been proposed as a future strategy. It may help identify patients with increased inflammatory activity associated with vulnerable plaques, may contribute to more refined patient selection for further diagnostic testing, and may support future personalized preventive strategies pending prospective validation. Furthermore, by differentiating inflammatory from purely ischemic chest pain syndromes, this approach may help avoid repeated emergency visits and diagnostic uncertainty in patients with non-obstructive CAD, ultimately improving both patient outcomes and healthcare efficiency. However, it should be emphasized that inflammation-based CT biomarkers such as PCAT-FAI are not currently incorporated into ESC, AHA, or ACC guideline recommendations for emergency chest pain triage.

## 8. Future Directions and Research Gaps

Despite substantial progress, several challenges must be addressed before PCAT-FAI and inflammation-focused imaging can be fully integrated into routine emergency care. A key priority is the standardization of PCAT and FAI acquisition, analysis, and reporting. Current variability in scanner types, reconstruction algorithms, and segmentation protocols limits cross-center comparability and hampers widespread adoption. International consensus on technical standards and quality control measures will be essential to ensure reproducibility and regulatory approval.

Despite growing evidence supporting PCAT-derived biomarkers, several technical factors may influence FAI quantification. Variability in scanner vendors, tube voltage settings, reconstruction kernels, and contrast acquisition protocols may affect perivascular attenuation values and limit direct comparability across centers. Although automated post-processing platforms have improved measurement reproducibility, interobserver variability and dependence on proprietary software remain important considerations. In addition, the integration of cloud-based AI platforms into routine clinical workflows raises practical issues related to cost, data governance, and regulatory approval.

Another important research direction is the formal integration of PCAT-FAI into emergency chest pain pathways. While accumulating evidence supports its prognostic value, prospective studies evaluating FAI-guided clinical decision-making in emergency departments remain limited. Randomized trials are needed to determine whether incorporating inflammation metrics into triage algorithms improves outcomes, reduces recurrent emergency visits, or optimizes resource utilization compared with standard care.

Multicenter validation in post-viral and systemic inflammatory states represents a particularly urgent research gap. The COVID-19 pandemic has highlighted the long-lasting cardiovascular consequences of viral infections, including persistent coronary inflammation and increased vulnerability to acute events even in patients without obstructive CAD. Whether similar inflammatory signatures occur after other viral illnesses, such as influenza, and how long these changes persist remain largely unknown. Large, longitudinal, multicenter studies are required to clarify the role of PCAT-FAI in these settings and to define appropriate surveillance and treatment strategies.

Finally, the development of AI-driven prediction models capable of estimating short-term ACS risk represents one of the most promising future directions. By integrating plaque morphology, PCAT radiomics, functional metrics such as CT-FFR, and clinical variables, machine-learning algorithms may enable real-time identification of patients at imminent risk of plaque rupture or arrhythmic events. Datasets such as ORFAN provide an unprecedented foundation for training and validating such models, but careful attention to transparency, explainability, and clinical interpretability will be essential to ensure safe implementation.

## 9. Conclusions

Coronary inflammation has emerged as a central determinant of plaque vulnerability and acute cardiovascular events. Among available imaging biomarkers, PCAT-derived fat attenuation index stands out as the most promising non-invasive biosensor of coronary inflammatory activity, capable of capturing dynamic biological processes that precede plaque destabilization. Unlike traditional anatomical markers, FAI provides real-time insight into disease activity and responds to therapeutic interventions, making it particularly relevant for both emergency care and longitudinal monitoring.

Incorporating inflammation-focused imaging into routine CAD assessment has the potential to fundamentally transform emergency cardiovascular care. By integrating CAC, CCT, and PCAT-FAI within a unified diagnostic framework, clinicians can move beyond the binary assessment of stenosis toward a more personalized, biology-driven evaluation of risk. As standardization improves and AI-enhanced analytics mature, this approach may enable earlier intervention, more precise triage, and ultimately better

## Figures and Tables

**Table 1:** CAD-RADS 2.0 plaque burden classification by CAC, SIS, and visual assessment [43].

Plaque Category	Total Amount of Plaque	CAC	SIS	Visual Estimation
P1	Mild	1–100	≤2	Mild plaque in 1–2 coronary vessels
P2	Moderate	101–300	3–4	Moderate plaque in 1–2 vessels or mild in 3 vessels
P3	Severe	301–999	5–7	Moderate plaque in 3 vessels or severe in 1 vessel
P4	Extensive	>1000	≥8	Severe plaques affecting multiple vessels

Abbreviations: CAC–coronary artery calcium score; SIS–segment involvement score.

## Data Availability

No new data were created or analyzed in this study. Data sharing is not applicable to this article.
